# Fostering informed empathy through patient-centred education about persons with disabilities

**DOI:** 10.1007/s40037-015-0197-5

**Published:** 2015-07-17

**Authors:** Sonya R. Miller

**Affiliations:** Departments of Physical Medicine and Rehabilitation and Learning Health Sciences, University of Michigan Medical School, 325 E. Eisenhower Parkway, 2nd Floor, 48108 Ann Arbor, MI USA

**Keywords:** Advocacy, Attitudes, Disability, Informed empathy, Patient-centred education

## Abstract

**Introduction:**

Having a disability can negatively affect provider-patient communication. Persons with disabilities report the need for better communication with their health care providers and argue that education regarding disabilities is lacking for health care professionals. We sought to determine if a patient-centred curriculum focused on individuals with disabilities could foster the development of informed empathy.

**Methods:**

An educational module to enhance health care students’ capacity for informed empathy was developed. To assess the development of informed empathy, a qualitative analysis of the post-module question, ‘How has your understanding, awareness or perception of individuals with disabilities changed?’ was performed.

**Results:**

Themes of the qualitative analysis were (a) becoming familiar with the daily life of individuals with disabilities, (b) changing notions of normalcy, (c) seeing discrimination against individuals with disabilities as an issue that impacted them, (d) recognition that disability is not only an issue of the physical body.

**Conclusions:**

Informed empathy can be effectively taught through a patient-centred curriculum focused on persons with disabilities. Health care providers are effective advocates when they understand the physical, emotional, social, and communication issues of persons with disabilities.

## Introduction

According to the 2013 American Community Survey, 12.6 % of the civilian, non-institutionalized population in the United States (approximately 39 million people) has a disability [1]. The Centers for Disease Control and Prevention estimates the prevalence of disability to be 20 % [2]. Having a disability has been negatively associated with provider-patient communication [3]. Persons with disabilities often perceive attitudinal and environmental barriers when trying to obtain health care [4, 5] and report the need for better communication with their health care providers [3, 4, 6]. Persons with disabilities argue that education regarding disabilities is lacking and that this is a major cause of miscommunication [4, 7, 8].

This perceived gap in health care provider education can be addressed through cultural sensitivity and disability awareness training [4, 6], but typically is not [9]. Teaching culturally competent care for persons with disabilities has to include their viewpoints and perspectives [9, 10]. Understanding some of the core values, beliefs, and experiences of persons with disabilities is essential to providers offering patient-centred care.

By increasing health care providers’ understanding of life with a disability, communication between persons with disabilities and providers may improve, which may foster greater satisfaction with care for all involved [11, 12].

Connor-Greene defines *informed empathy* as the blending of knowledge about mental illness with an appreciation of its personal impact on individuals, their families, and those who provide their care [13]. Teaching informed empathy encompasses helping students understand not only factual information but also what it might feel like to have a specific disorder. Consequently, the development and use of informed empathy is an avenue by which patient-centred care and patient-centred education can be enhanced.

We adapted the definition of informed empathy by Connor-Greene to include knowledge about the impairments, activity limitations, and participation restrictions that are associated with having a physical disability, blended with an appreciation of the personal impact these issues have on individuals, their families, and those who provide their care [13, 14]. A patient-centred educational module specifically focused on persons with disabilities was developed to help current and future health care providers better understand some of the realities and complexities of living with a disability, as well as increase their knowledge about issues that are relevant and important to this community [3]. This study is a qualitative assessment of the quantitative study performed on this patient-centred curriculum to foster the development of informed empathy [15].

## Methods

This study was approved by the medical school’s institutional review board.

### Curriculum development

The curriculum was designed to evoke reflections about attitudes, empathy, and the role of advocacy for health care professionals. To ground the educational experience in authentic representation of patients’ experiences, we developed a DVD specifically for this curriculum [15]. The DVD consists of narratives by and about persons with disabilities. A total of 11 men and 7 women, age 21–72, with a variety of diagnoses including spinal cord injury, lower extremity amputation, peripheral neuropathy, blindness, vasculitis, and cancer were recruited.

Participants were asked to share experiences or other information they wanted current or future health care professionals to know. Based on their preference for writing or using a micro-cassette recorder, participants provided written or recorded narratives about their lives and health care experiences. They were encouraged to provide an artistic interpretation (e.g., a drawing, a poem, or photographs) of their experiences. After reviewing all of the narratives, additional conversations were conducted to ascertain the most salient topics for the participants.

A 60-minute DVD containing an oral summary of 18 narratives was created. Each narrative is linked to one or more images. In some cases, the image is a photo, drawing, or collage provided by the individual.

### Curriculum implementation

The principal researcher taught the module in an upper level undergraduate health-related course at the end of the term. This module was the only curriculum content about the psychosocial aspects of disability.

The module comprised 3 h over two sessions. To build trust and create a safe environment for sensitive discussions, students were initially asked low risk questions related to the type of health care provider they plan to become and possible practice setting. Next, students discussed definitions including but not limited to disability, health, and advocacy. Students were asked about their experiences with persons with disabilities and advocacy. It was stressed that the narratives were in the speakers’ own words and the concepts were those the speakers wanted to share with health care providers.

In each session, students viewed multiple segments of the DVD. After watching 7–10 min of the DVD, a discussion was initiated by asking fundamental questions, such as, ‘Which reaction/response did you understand the most or least? Which accommodations are reasonable and how much is enough?’ Students formed groups of two or three and discussed the questions and their impressions. Then, the class came back together and each group gave a summary of its discussion. This led to a more detailed, in-depth discussion of a topic involving multiple members of the class. Then, additional segments of the DVD were viewed and the above process was repeated.

### Students

Forty-one undergraduate students enrolled in a health-related course participated in the study. Students’ answers were not matched with any demographic data. Approximately 2 weeks after the module was taught, students were asked, ‘As a result of participating in the class sessions entitled ‘Aspects of Life with a Disability’, how has your *understanding, awareness or perception* of individuals with disabilities changed?’ We qualitatively assessed the students’ written responses using a semi-grounded approach to determine the most common themes.

## Results

NVivo 10 was used to develop a coding scheme and to code the responses. The identification of four themes and specific quotes (Table 1) suggests the students were developing informed empathy, blending knowledge about physical disabilities with an appreciation of its personal impact on individuals and their families.

**Table 1:** Identified themes from student responses

Theme	Quote
Becoming familiar with the daily life of individuals with disabilities	‘It has really opened my eyes to the perspectives of the individuals themselves. It is hard to imagine what life is like with a disability until one is actually acquired, but the stories and commentaries form the DVD were eye opening to life with a disability.’ ‘However I did not realize how strongly of an effect it had on their energy [*sic*] day lives and how much of a social aspect was considered.’ ‘I would sometimes feel sorry for those with disabilities but have realized that is not what they want. I learned they want to be treated as individuals and want to be as independent as they can. I will make sure to respect the wishes of an individual with a disability and not try to judge what they want just based on their condition.’
Changing notions of normalcy, in which persons with disabilities consider themselves normal	‘My awareness has increased about how individuals with disabilities view themselves and don’t want to be treated any differently from someone else.’ ‘My awareness of those with disabilities has changed in that they want to be seen as a normal person, and not someone who is incapable and disabled.’
Seeing discrimination against individuals with disabilities as issues that impacted them	‘With or without a disability, anyone is the same person. The disability doesn’t define you or necessarily what you are capable of accomplishing in your life.’
Recognition that disability is not only an issue of the physical body	‘Hearing the social and emotional challenges was very new to me and I learned a great deal from it.’

1. Students learned how lack of accessibility and distorted or inaccurate perceptions of disability are daily challenges for persons with physical disabilities. They realized that persons with disabilities can be offended when others automatically assume they need assistance. Students recognized that persons with disabilities want to be independent and treated with the same respect as persons without disabilities; they do not want to be judged based on their disability.
2. Students changed their notions of normalcy and began to understand how persons with disabilities consider themselves normal. Individuals with disabilities want others to interact with them in the same way they interact with non-disabled individuals. Students remarked that persons with disabilities want a quality of life similar to persons without disabilities.
3. Students discovered that persons with disabilities endure many forms of discrimination including environmental barriers, social stigmas, negative connotations regarding their lives, the tendency to underestimate their quality of life and disparities in health care.
4. Students expressed an understanding that disability is not limited to the physical body, but also includes social, emotional and communication issues. Students remarked that disability status can influence and change perceptions and acknowledged a better understanding of the complexity of disabilities. They commented on how persons with disabilities can be stereotyped based solely on their disabilities and how society’s perception of a person can be altered and influenced by whether or not a disability is present.

## Discussion

This curriculum is focused on patient-centredness and patient-centred education. The patients are the educators. The curriculum focuses on ideas, concepts and experiences that persons with disabilities feel are important. The students gained an understanding of the challenges persons with disabilities face on a daily basis including physical barriers. Some students expressed an increased awareness of how they interact with persons with disabilities. There was an appreciation of the myriad of ways normalcy can be defined, including having a disability. The students commented on the various forms of discrimination faced by individuals with disabilities such as environmental and social barriers, or social stigma and how well-being can be affected. There was also acknowledgement that a person’s disability does not have to be ‘fixed’ and that individuals can have disabilities and be happy with themselves and their lives.

The small sample size limits the generalizability of the results. However, our understanding of how the students understand and view persons with disabilities and conceptualize empathy is enriched. This module is easily incorporated into existing course curricula, requiring a maximum of 3 h teaching time.

Utilizing a module based on patient narratives and their experiences allows students to better understand patient perspectives. This may contribute to optimal communication and greater trust between the individuals with disabilities and their health care providers, which may foster greater satisfaction with care for all involved. In addition, the overall quality of care may improve.

No longitudinal effects of the module were assessed. Ideally, these students would be monitored after completing their training and entering practice to determine if their clinical outcomes, professionalism and communication skills differ from their peers who did not receive this training.
